# Dysregulated Peroxiredoxins in Bladder Cancer Are Associated With an Altered Tumour Immune Microenvironment

**DOI:** 10.1111/jcmm.71064

**Published:** 2026-02-27

**Authors:** Jarett Wallerson, Sara Rostampour, Shaolei Teng, Dawit Kidane

**Affiliations:** ^1^ Department of Physiology & Biophysics, College of Medicine Howard University Washington DC USA; ^2^ Department of Biology Howard University Washington DC USA

## Abstract

Peroxiredoxins (PRDXs) are antioxidant enzymes that scavenge hydrogen peroxide and protect cells from reactive oxygen species (ROS). There are six genes encode different types of PRDXs (PRDX1–PRDX6) in humans and most of them are overexpressed in tumours; however, their expression patterns and prognostic value in bladder cancer (BLCA) remain unclear. In this study, we examined the aberrant expression of all six types of PRDX genes in BLCA and identified distinct clinical and immune associations. High expression of PRDX1 and PRDX6 was correlated with poor overall survival (OS), increased mutational burden and chromosomal instability. Overexpression of PRDX4 and PRDX6 was associated with advanced tumour stage, larger tumour size, higher immune scores, and increased immune cell infiltration. By contrast, PRDX2 overexpression showed only modest effects on OS and was associated with reduced immune signalling and diminished infiltration of anti‐tumor immune cells. These findings highlight the differential roles of PRDX family members in shaping BLCA tumour immune microenvironment. PRDXs may serve as prognostic biomarkers for patients tratification and represent potential therapeutic targets to enhance immunotherapy response. Further in vitro and in vivo studies are required to confirm our in silico data and define their clinical relevance for BLCA prognosis.

## 1 Introduction

Bladder cancer is the sixth most prevalent cancer type in the United States, with an estimated 83,190 new cases reported in 2024 [1]. Urothelial carcinoma, referred to as BLCA in the Tumor Cancer Genome Atlas (TCGA) is the predominant histologic subtype analyzed in this study [2]. The majority of patients are diagnosed with non–muscle‐invasive bladder cancer (NMIBC) with 10% to 20% of cases progressing to muscle‐invasive or metastatic disease [3]. Despite different therapeutic approaches, the overall survival (OS) of BLCA patients remains low [2, 4]. The 5‐year survival rate is approximately 77% and < 15% for those with metastatic disease [3]. To improve survival and quality of life for BLCA patients, it is critical to unravel the mechanisms of how PRDXs antioxidant enzymes in BLCA cells modulate oxidative stress‐associated genomic integrity and impact the tumor immune microenvironment.

Cancer cells often have high levels of ROS, such as superoxide anion, which can lead to oxidative DNA damage [5]. Thus, cancer cells must maintain adequate levels of antioxidant defences (e.g., GSH levels, PRDXs, reduced CoQ_10_) for growth and survival [6, 7]. Recent studies demonstrated that proliferation of cancer cells leads to the production of elevated ROS levels due to increased metabolic demand and modified antioxidant defence systems [8]. Oxidative stress is one of the main features of cancer cells and is caused by an imbalance between ROS production and antioxidant defences. ROS contribute to nuclear genome (nDNA) or mitochondrial deoxyribonucleic acid (mtDNA) mutations that promote tumour initiation and progression [9]. Whereas, antioxidants participate in neutralising ROS‐induced cellular macromolecule damage, which may facilitate tumour growth and treatment resistance. Genomic analysis demonstrated that BLCA tumours harbour loss of tumour suppressor genes (e.g., p53, CDKN2A) [10], chromatin remodelling complex modifier (AT‐rich interaction domain 1A [ARID1A]) [11, 12, 13] and mutations of the DNA damage and repair genes that likely synergise ROS‐induced genomic instability [14, 15].

BLCA cells overexpressed an antioxidant to control the levels of ROS [16, 17]. PRDXs enzymes are cysteine‐dependent peroxidases that are responsible for the reduction of more than 90% of intracellular peroxide in humans [18, 19, 20]. Apart from its canonical role in regulating of redox signalling such as reducing hydrogen peroxide, peroxynitrite and other hydroperoxides [18, 21, 22, 23]. PRDXs are involved in the regulation of cell growth, metabolism, hormone signalling, immune regulation [24, 25]. PRDX is overexpressed in multiple cancer types, including BLCA and contributes significantly to carcinogenesis [26].

Therefore, uncovering the biological significance of altered expression of PRDXs in bladder tumours likely fills the knowledge gap and uncover the different aspects of the tumour immune microenvironment dynamics. Here, we investigated the PRDXs transcriptional change in bladder tumour their impact on genomic stability and innate immune signalling to pinpoint the immunogenicity of tumour immune microenvironment in BLCA. Our in silico data show that approximately 29% of tumours overexpressed PRDXs (TCGA data) and PRDX gene amplification and deletion estimated to a range between 1% and 7% of the tumour. Additionally, patients with tumours that have high PRDX1 and PRDX6 expression have significantly lower overall survival (OS) rates compared to patients whose tumour expresses lower levels of PRDX1 and PRDX6. Furthermore, selective types of PRDXs overexpressing bladder tumours harbour greater genomic instability. Moreover, tumours with overexpressed PRDX2, PRDX3 and PRDX5 harbour low tumour immunogenicity as indicated by poor anti‐tumour immune cell infiltration and innate immune signalling. Finally, our study showed a negative correlation between PRDX2, PRDX3 and PRDX5 expression in bladder tumours and tumour immune checkpoint expression and immune cell infiltration in the tumour microenvironment. While the biological relevance of the correlations is unclear, it raises the possibility that this profile would help explain the worse prognosis observed in PRDXs‐overexpressing bladder cancer patients. Furthermore, these findings have clinical implications for bladder cancer patients for whom PRDXs should be explored as a potential prognostic marker to stratify patients for future immune‐based therapeutic strategy in BLCA.

## 2 Material and Methods

### 2.1 Data Acquisition

Patient tumour RNA sequencing (RNA‐seq) data and patients' clinical history including tumour histological grade, survival status and tumour subtype, tumour size, tumour stage and lymph node status were retrieved (n=408) from the Cancer Genome Atlas [TCGA Pan‐Cancer: cBioPortal (http://www.cbioportal.org)]. Patients' data were categorised as PRDXs with high and low expression to analyse the tumour data, including the tumour stage, lymph node status, histological grade and patient characteristics as described in our previously published data analysis [27].

### 2.2 Exclusion and Inclusion Criteria

Patients' tumour samples with valid RNA‐Seq V2 RSEM data for PRDX1–6 were included. Patients were categorised into expression groups using *z*‐scores value: those with *z*‐scores < −0.5 were considered low‐expressed group, and those with *z*‐scores > 0.5 categorised as the high‐expression group. Patients' RNA‐Seq expression *z*‐score range between −0.5 ≤ *z* ≤ 0.5 were excluded from data analysis to enhance the contrast between high‐ and low‐expression groups.

### 2.3 Evaluating Immune Cell Infiltration, Estimation of Immune and Stroma Score in Tumour Tissues

The TIMER algorithm was applied to evaluate the immune cell infiltration in the tumour microenvironment. Immune cells including CD4+ T cells, CD8+ T cells, B cells, neutrophils, macrophages and dendritic cells were selected, and compared their immune cell infiltration correlation with RNA‐Seq expression levels of PRDX1‐PRDX6. We applied Spearman correlation analysis. Furthermore, we used MD Anderson cancer centre ESTIMATE algorithm‐generated matrix, to calculate the immune score and stroma score at the tumour microenvironment.

### 2.4 Mutation Count and CNA

BLCA patient tumour sample mutation counts were obtained from the TCGA dataset. The mutation count values were grouped according to low and high PRDXs (PRDX1‐6) expression levels. We analysed the data using an unpaired *t*‐test to assess the statistical differences between the two groups. Further, BLCA patient tumour samples with CNA data also collected from the cBioPortal website and merged with the TCGA data set and analysed using unpaired *t*‐test to obtain the difference between PRDX1‐6 low and high expression groups.

### 2.5 Statistical Analysis

We applied Spearman rank correlation test to analyze the immune cell infiltration and expression of RNA‐seq expression data. Kaplan–Meier analysis was used to estimate the overall survival. Univariate associations between PRDXs overexpression and clinicopathologic variables were tested using nonparametric tests. Statistical significance was set at *p* < 0.05. Further, the prognostic value of PRDX expression in tumour versus normal tissues was analysed using the ROC of the GraphPad Prism software.

## 3 Results

### 3.1 PRDXs Dysregulated in BLCA

**FIGURE 1:**
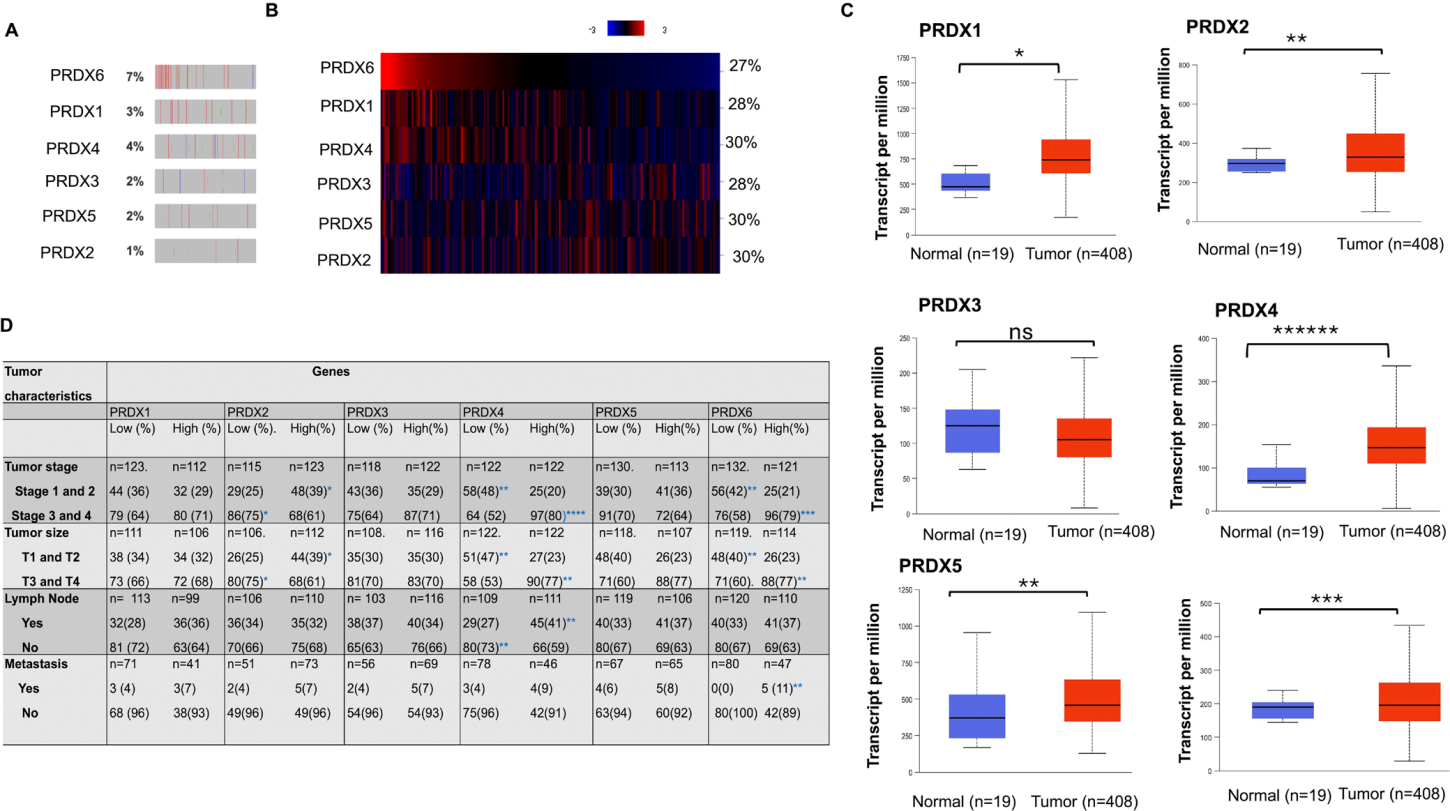
Expression and genomic alterations of PRDX family members in bladder cancer. (A) Copy number alterations of PRDX1–6 in BLCA patients from TCGA. Percentages indicate the frequency of amplification or deletion. (B) Heatmap of PRDX1–6 mRNA expression in BLCA tumours showing the proportion of cases with high expression. (C) Comparison of transcript levels (TPM) for PRDX1–6 between normal bladder tissues (*n* = 19) and BLCA tumours (*n* = 408). PRDX1, PRDX2, PRDX4, PRDX5 and PRDX6 are significantly upregulated in tumours, whereas PRDX3 shows no significant difference. (D) Relative expression of PRDX1‐PRDX6 stratified by tumour stage (I–IV) and tumour size (T1–T4). Overexpression is significantly higher in late‐stage (III–IV) and larger tumours (T3–T4). Statistical significance for tumour versus normal tissues (C) and stage/size comparisons (D) was determined using unpaired *t*‐tests or Wilcoxon rank‐sum tests as appropriate; *p < 0.05, **p < 0.01, ***p < 0.001, ****p < 0.0001, *****p < 0.00001 ns = not significant.

To examine whether PRDXs gene integrity in tumour samples, we analysed TCGA bladder urothelial carcinoma (BLCA) cancer data set and found that the PRDXs gene amplification and deletion ranges from 1% to 7% (PRDX1 to PRDX6 respectively; Figure 1A). Additionally, abnormal expression of mRNA of PRDXs found that approximately in 30% of the tumours (Figure 1B). Further, the UALCAN database was used to investigate the link between PRDXs gene expression between tumour versus normal tissues. Our results illustrated that PRDX1, PRDX2, PRDX4, PRDX5 and PRDX6 had a significantly higher expression level at all stages of tumour than in normal tissues (Figure 1C). However, no significant differences in PRDX3 expression were observed at the transcript level between tumour versus normal samples. In addition, the mRNA expression of PRDX4 and PRDX6 overexpression significantly higher in stage III and IV as well as tumours size T3 and 4, as compared with tumours that harbour low PRDX4 and PRDX6 expressed tumour tissues (Figure 1D). Moreover, as shown in Figure 1D, the expression patterns of PRDX1, PRDX2 and PRDX5 were enhanced in all BLCA, whereas no statistical differences in PRDX3 expression were observed between BLCA and normal bladder tissues. We have also noticed that a moderate increase in PRDX3 and PRDX1 overexpression is associated with tumour stage III and IV, lymph node and metastasis. In addition, PRDX6 mRNA expression in BLCA metastasis was higher than that in low‐expressing tumour samples (Figure 1D; **p < 0.01). These findings demonstrate that PRDX family members play important roles in BLCA cancer progression.

### 3.2 Altered Levels of PRDXs Are Associated With Poor Overall Survival of BLCA Patients

**FIGURE 2:**
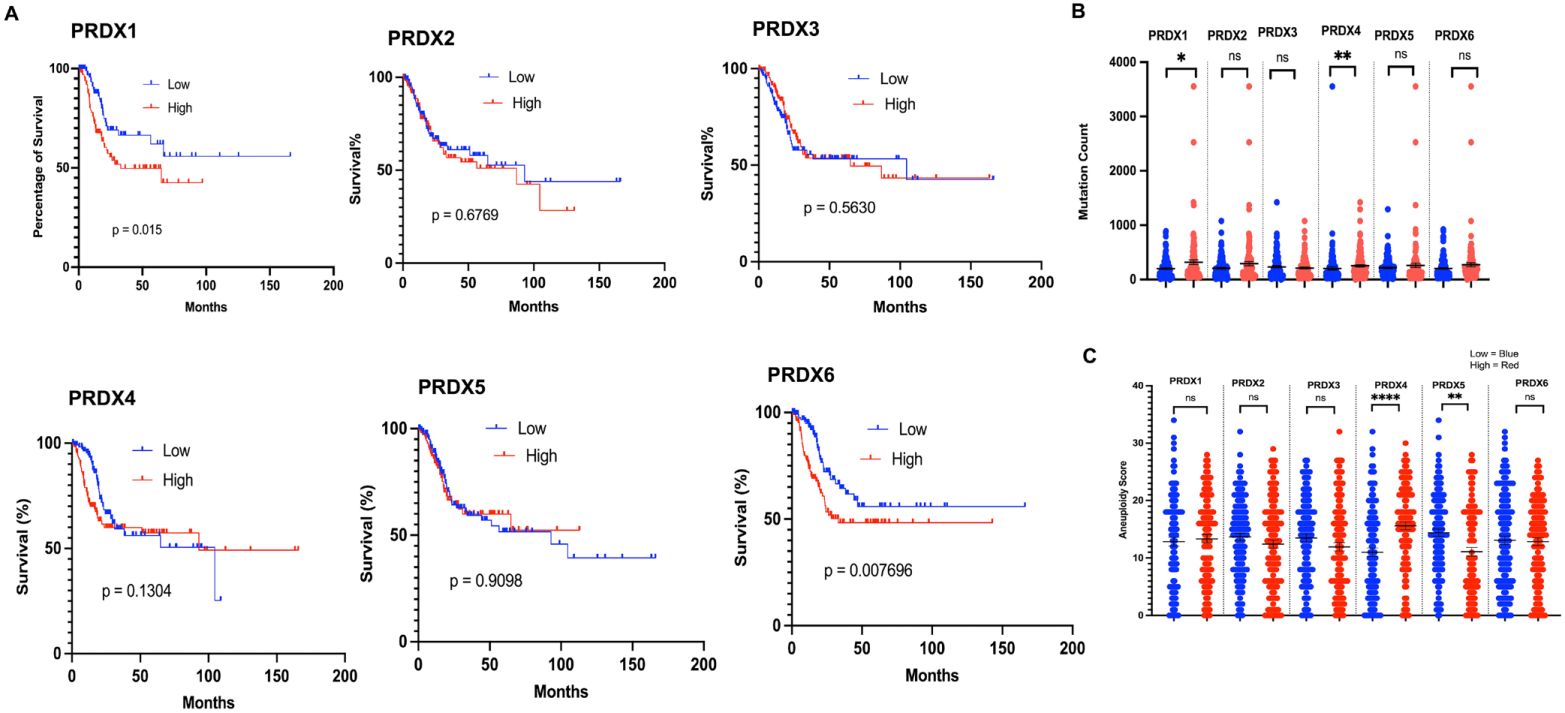
Association of PRDX expression with overall survival and genomic instability in bladder cancer. (A) Kaplan–Meier survival curves showing the relationship between PRDX1–6 expression and overall survival in BLCA patients (TCGA dataset). High expression of PRDX1 and PRDX6 is associated with significantly worse survival, whereas PRDX2, PRDX3, PRDX4 and PRDX5 show no significant differences. (B) Comparison of mutation counts between tumours with high and low PRDX expression. Tumours with high PRDX1 and PRDX6 expression exhibit significantly higher mutation burdens. (C) Aneuploidy scores in high versus low PRDX expression groups. Overexpression of PRDX4 and PRDX5 is associated with significantly higher aneuploidy compared with low‐expression tumours. Statistical significance for survival analyses (A) was determined using the log‐rank test, and for mutation counts and aneuploidy scores (B, C) using unpaired *t*‐tests; *p < 0.05, **p < 0.01, ****p < 0.0001.

PRDX1 and PRDX6 were significantly overexpressed and associated with poor overall survival (Figure 2A; *p < 0.01 and **p < 0.0001). However, no significant difference was observed between tumours with overexpression of PRDX2, PRDX3, PRDX4 and PRDX5 compared with low expression of PRDXs in tumours (Figure 2A). Further, we examined whether overexpression of PRDXs genes was associated with genomic instability in BLCA. The number of mutation counts were significantly increased in PRDX1 and PRDX6 overexpressing tumours of BLCA in comparison to low PRDX1 and PRDX6 expressing tumours (Figure 2B; ***p < 0.001). Importantly, PRDX4 and PRDX5 overexpression was associated with a higher aneuploidy scores than BLCA patients with low PRDX4 and PRDX5 expressions (Figure 2C; ***p < 0.001).

### 3.3 Overexpression of PRDXs in Tumour Is Associated With Dysregulated Immune Score and Immune Cell Infiltration

**FIGURE 3:**
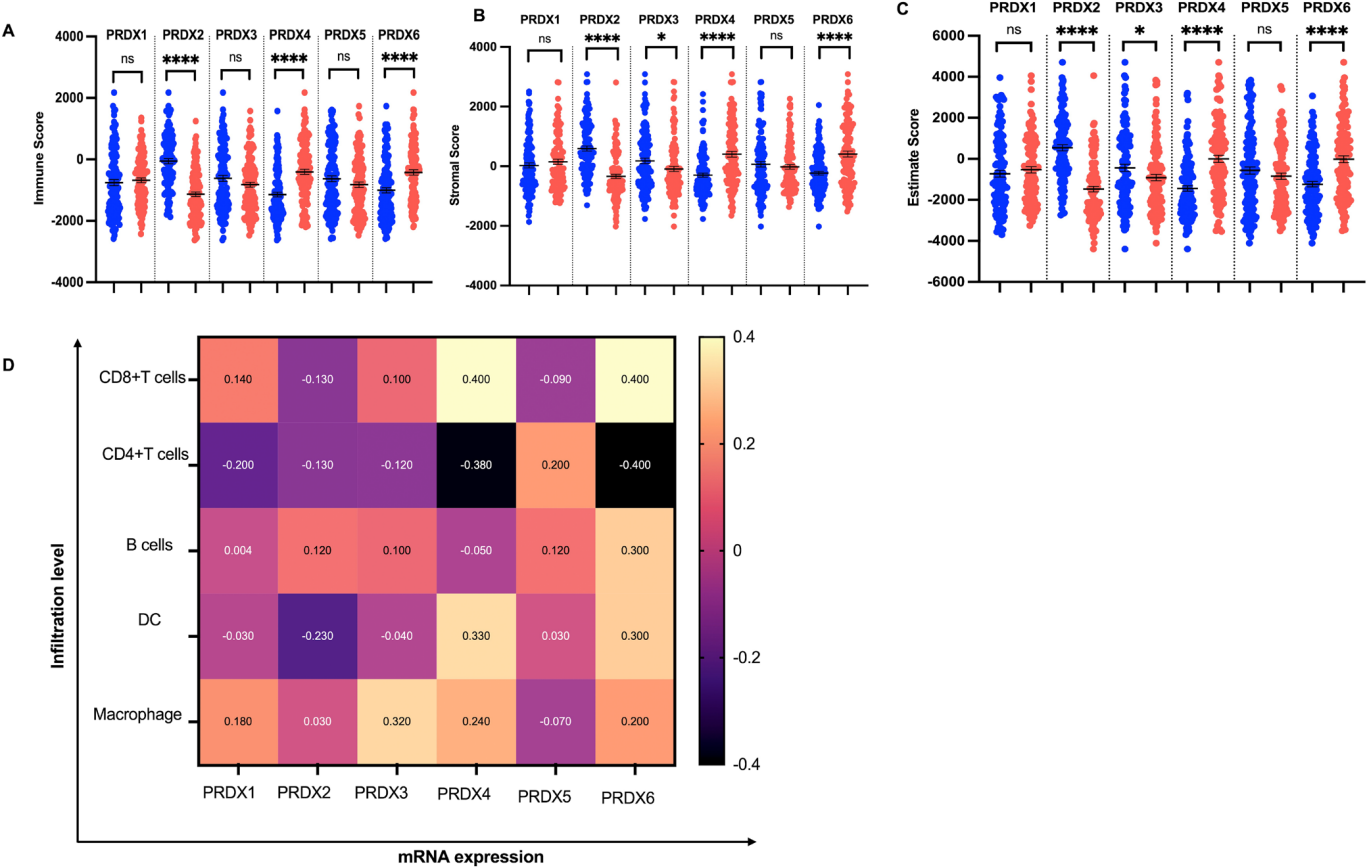
Relationship between PRDX expression and the tumour immune microenvironment in bladder cancer. (A–C) ESTIMATE analysis of immune, stromal and ESTIMATE scores in tumours with low versus high PRDX expression. High PRDX2 expression is associated with significantly lower immune and stromal scores, whereas high PRDX4 or PRDX6 expression corresponds to significantly higher scores. (D) TIMER‐based analysis of immune cell infiltration. PRDX2‐overexpressing tumours exhibit reduced infiltration of CD8^+^ T cells, CD4^+^ T cells and B cells, whereas PRDX4 and PRDX6 overexpression is associated with increased infiltration of CD8^+^ T cells, dendritic cells and macrophages. Spearman correlation coefficients are indicated. Statistical significance: ****p < 0.0001, *p < 0.05.

Several studies have suggested that the presence or absence of immune cells within the tumour microenvironment determines the outcomes of cancer treatment [28, 29, 30]. We compared the immune and stromal scores between low and high PRDXs expressing tumour samples and found that PRDX2 overexpressing tumours were significantly associated with lower immune scores, stromal scores and ESTIMATE scores (Figure 3A–C). Intriguingly, we performed a comprehensive investigation of the correlations between the expression levels of PRDX2 and immune cell infiltration on tumours using the TIMER database. High PRDX2 expression was associated with a reduced innate and adaptive immune cell abundance in tumour which included CD8+T cells (correlation = −0.14), CD4+ T cells (correlation = −0.13), B cells (correlation = 0.12), In contrast, overexpression of PRDX4 and PRDX6 in tumour harbour was associated with significantly higher immune scores, ESTIMATE score as compared with low PRDX4 and PRDX6 expressing tumour (Figure 3A–C; ****p < 0.0001; **p < 0.001). Further, positive correlation with CD8+ T cells (correlation = 0.4), dendritic cells (correlation = 0.33), macrophages (correlation = 0.23) and in contrast with negative correlation with CD4+ T cells (correlation = −0.38) (Figure 3D). These results showed that selective types of PRDX2 gene expression associated with immune‐deserted tumour microenvironment. In contrast, PRDX4 and PRDX6 play a significant role in enhanced anti‐tumour immune infiltration to modulate the tumour microenvironment.

### 3.4 Association of PRDXs With Innate Immune Signalling Landscape and Immune Checkpoint Genes in BLCA

**FIGURE 4:**
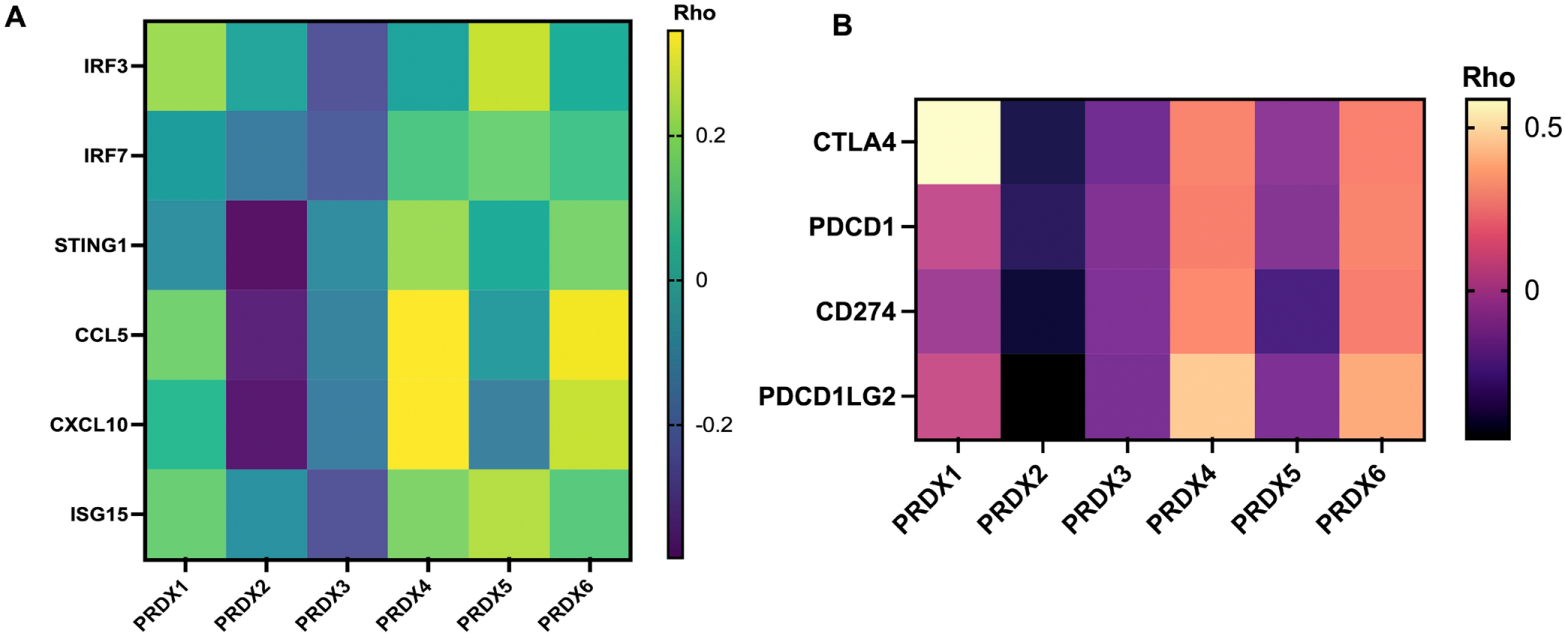
Correlation of PRDX expression with innate immune signalling and immune checkpoint genes in bladder cancer. (A) Correlation between PRDX1–6 expression and innate immune signalling genes (CCL5, CXCL10, ISG15, STING1). Overexpression of PRDX2, PRDX3 and PRDX5 is associated with reduced expression, whereas PRDX4 and PRDX6 overexpression correlates positively with higher expression levels. (B) Heatmap of immune checkpoint gene expression (CTLA4, PDCD1, CD274, PDCD1LG2) in tumours with high versus low PRDX expression. High PRDX2, PRDX3 and PRDX5 expression shows negative correlations with checkpoint genes, whereas PRDX4 and PRDX6 show positive correlations. PRDX1 exhibits mixed correlation patterns, including a positive association with CTLA4. Correlations were calculated using Spearman analysis; ***p < 0.001, ****p < 0.0001.

We examined whether an altered levels of PRDX expression is associated with innate immune signalling genes expression and immune checkpoint mediator genes. We performed a Spearman correlation analysis on BLCA with low and high PRDXs expression using the publicly available cancer genomic database, including TCGA Pan‐Cancer dataset. We found that BLCA with high PRDX2, PRDX3 and PRDX5 have low expression of innate immune signalling genes (Figure 4A; CCL5, CXCL10, ISG15, STING1). In contrast, the PRDX4 and PRDX6 overexpression results indicate a positive correlation with the expression of CCL5 and CXCL10 (Figure 4A, ***p < 0.001). Furthermore, our study examined whether PRDXs expression in BLCA is associated with altered expression of immune checkpoint genes (CTLA4, PDCD1, CD274 and PDCD1LG2) (Figure 4B). Heatmap of gene expression analysis shows that the expression of PRDX2, PRDX3 and PRDX5 is significantly negatively correlated with all CTLA4, PDCD1, CD274 and PDCD1LG2 in BLCA (Figure 4B). In particular, tumours with high PRDX2 expression were significantly associated with low expression of immune checkpoint genes (Figure 4B; range −0.3 to −0.4). In contrast, PRDX4 and PRDX6 overexpressing tumours harbour more expression of checkpoint genes (Figure 4B; range of correlation = 0.25–0.45). In addition, as shown in Figure 4B, overexpression of PRDX1 has a negative correlation with the expression of the majority of the checkpoints except the CTLA4 gene (correlation = 0.56).

### 3.5 PRDXs Have a Prognostic Value for BLCA

**FIGURE 5:**
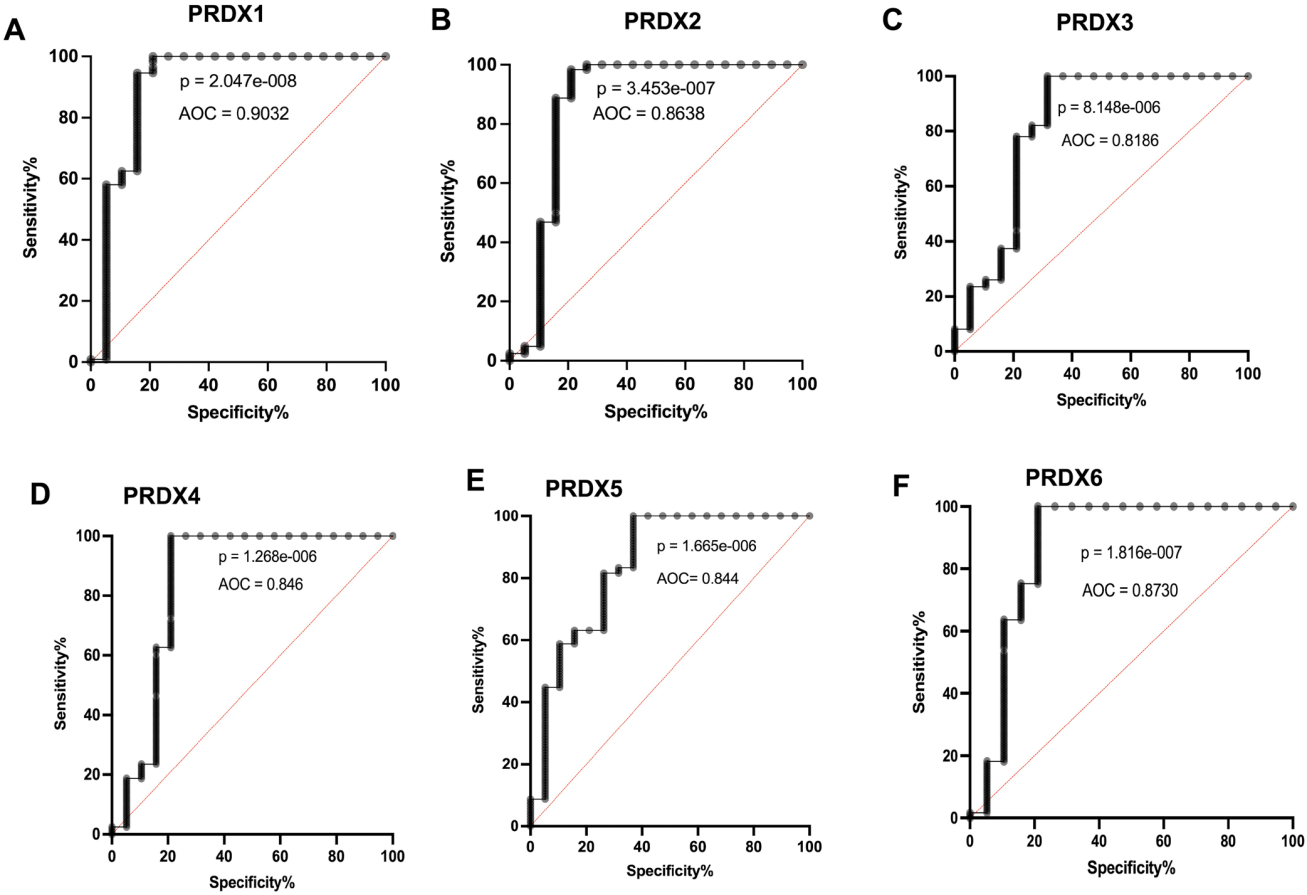
Prognostic value of PRDX family members in bladder cancer. Receiver operating characteristic (ROC) curve analysis of PRDX1–6 expression in BLCA. (A) PRDX1 (AUC = 0.90), (B) PRDX2 (AUC = 0.86), (C) PRDX3 (AUC = 0.81), (D) PRDX4 (AUC = 0.85), (E) PRDX5 (AUC = 0.84) and (F) PRDX6 (AUC = 0.87) show significant predictive ability to distinguish BLCA tumours from normal tissues. Area under the curve (AUC) values with 95% confidence intervals (CIs) are indicated. Statistical significance was determined using ROC analysis; ****p < 0.0001.

To examine the diagnostic value of PRDX genes in BLCA, we performed ROC curve analysis. We calculated the area under the curve (AUC) with 95% confidence interval (CI) and found that the AUC was significantly high in tumours with overexpression of PRDX1 (AUC=0.90) (Figure 5A; 95% CI: 0.795–1.000; ****p < 0.0001) and PRDX6 (87%) (Figure 5F; 95% CI: 0.7451–1.000 ****p < 0.0001). Eighty six percent of AUC were calculated for PRDX2 overexpressed tumour with poor immunogenicity (Figure 5B; 86%, 95% CI 0.7250–1.000; ****p < 0.0001), In addition, the AUC for other tumour with high PRDXs expression including PRDX3 (Figure 5C; 81%, 95% CI: 0.6780–0.9591, ****p < 0.0001) PRDX4 (Figure 5D; 85%, 95% CI: 0.7037–0.9882 ****p < 0.0001) PRDX5 (Figure 5E; 84%, 95% CI: 0.7291–0.9588; ****p < 0.0001) were significantly high.

## 4 Discussion

Bladder cancer is a prevalent cancer worldwide and represents a significant health concern. Experimental evidence shows that cancer development is a multistage process and is associated with dysregulation of several types of genes [31]. In this part of our study, we investigated the association of the antioxidants encoding genes that likely protect the cancer cells from oxidative stress and their impact on patients' tumour immune microenvironment. We explored the expression of each member of the PRDXs family in BLCA at the transcriptional level. We found that the mRNA levels of PRDX1, PRDX2, PRDX4, PRDX5 and PRDX6 were significantly higher in tumour tissues than in normal tissues. Results from this study demonstrated that PRDXs are highly expressed in BLCA tumour tissues associated with key factors that likely modulate the redox balance in cancer cells to promote tumorigenesis through multiple pathways. Previous mechanistic studies demonstrated that highly expressed PRDXs can metabolise hydrogen peroxide to maintain the cancer cells survival [32]. From the perspective of molecular mechanisms, PRDXs in BLCA may play a similar role in maintaining the tumour microenvironment by modulating intracellular redox homeostasis which favours tumour cell proliferation and survival [33]. Additionally, high expression of PRDX1 and PRDX6 is also associated with poor overall survival in patients, late stage of tumour and tumour size, suggesting that selective groups of the PRDX family have a potential for prognostic assessment of bladder cancer patients. Our results are in agreement with Cha et al. [34], finding that tumours with high PRDX1 expression positively correlated with tumour grade. This suggests that selective PRDXs could be a potential prognostic biomarker for BLCA. However, we acknowledge that PRDX1, PRDX6 and PRDX4 may need further mechanistic investigation to uncover their role in BLCA.

The tumour microenvironment harbours dynamic heterogeneity in numbers and types of immune cells that determine the immunogenicity of the tumour [35]. Herein, our findings demonstrated that high infiltration of CD8+ T cells and dendritic cell (DC) abundance was remarkably correlated with a better overall survival. Prognostic or predictive biomarkers related to the tumour immune microenvironment may provide an advantage to identify novel molecular targets and improve immune‐based therapy. Our results show that the expression of mRNA of PRDX1, PRDX4 and PRDX6 demonstrated a significantly positive correlation with immune score, innate immune signalling (STING1, CXCL10, CCL5 and ISG15) gene expression and infiltration of immune cells, including CD4+ T cells and DC cells, suggesting a potential association with the anti‐tumour immune microenvironment. This suggests that the immune cell infiltration and innate immune signalling may enhance a better clinical outcome for patients that undergo immune‐based strategies. In contrast, PRDX2 overexpression in tumours significantly harbours low immune score, low innate immune signalling and poor immune cell infiltration that may favour tumour progression and may cause poor immune‐based therapy response. These data suggested that the immune‐desert phenotype was characterised by the absence of infiltrating immune cells and weakened immune activity in PRDX2‐overexpressed BLCA. Results from this work suggested that PRDX1, PRDX4 and PRDX6 are associated with an immunogenic tumour microenvironment.

Currently, several clinical data have pointed out a correlation between the genetic alterations and responsiveness to the immunological treatment [36, 37]. Results from this work pointed out overexpression of PRDX1 in tumour co‐occurred with low expression of the majority of the checkpoint genes except CTLA4. Our data suggested that those patients may be stratified for anti‐CTLA4‐based immunotherapy. In addition, tumours with overexpression of PRDX4 and PRDX6 co‐occurred with high expression of most checkpoint genes, which may open more opportunities for immune‐based therapy. In contrast, PRDX2, PRDX3 and PRDX5 expression in tumours has limited or no expression of checkpoints that likely impaired targeting those checkpoint receptors. This observation also may serve to minimise unnecessary immune‐based treatment and may mitigate toxicity during treatment. Therefore, our study suggested that selecting patients based on expression of PRDXs of different families may provides an alternative platform to strategise immune‐based therapy. Further, the addition of immune maintenance therapy to first‐line chemotherapy to stimulate PRDX2, PRDX3 and PRDX5 overexpressing tumours may play a role in enhancing immunogenicity by indicating targetable PD‐L1 expression similar to other types of tumours [38].

Overall, our study provides insights into the immune‐genomic dynamics of BLCA by examining PRDX family expression and their relationship with the tumour immune microenvironment. Our findings suggest that PRDX1, PRDX4 and PRDX6 may serve as potential prognostic markers and therapeutic targets for bladder cancer. However, the molecular mechanisms underlying the interactions among PRDX family members and their impact on bladder tumour progression remain to be fully elucidated. Future in vitro and in vivo studies integrating diverse biological pathways and cellular processes may offer a more comprehensive understanding of PRDX‐mediated regulation in BLCA and support the development of targeted therapeutic strategies.

## Author Contributions

D.K.: conceptualisation, funding acquisition, investigation, project administration, supervision, validation, writing – review and editing. J.W.: data curation, formal analysis, investigation, methodology, validation, writing – original draft, writing – review and editing. S.R.: data analysis and collecting Data. S.T.: Data analysis and writing manuscript.

## Funding

Research reported in this publication was supported by the National Cancer Institute (Grant R21CA249346) and the National Institute of Allergy and Infectious Diseases of the National Institutes of Health (Grant R01AI179899).

## Ethics Statement

The authors have nothing to report.

## Consent

The authors have nothing to report.

## Conflicts of Interest

The authors declare no conflicts of interest.

## Data Availability

All extracted data from cBioPortal (www.cBioPortal.org) and The Cancer Genome Atlas data sets used and/or analysed during the current study are available from the corresponding author on reasonable request.
